# An Enhanced Error Model for EKF-Based Tightly-Coupled Integration of GPS and Land Vehicle’s Motion Sensors

**DOI:** 10.3390/s150924269

**Published:** 2015-09-22

**Authors:** Tashfeen B. Karamat, Mohamed M. Atia, Aboelmagd Noureldin

**Affiliations:** Electrical and Computer Engineering, Royal Military College of Canada, Kingston, ON K7K 7B4, Canada; E-Mails: mohamed.atia@rmc.ca (M.M.A.); Aboelmagd.Noureldin@rmc.ca (A.N.)

**Keywords:** inertial sensors, wheel rotation sensors, Global Positioning System, gyroscope, accelerometer, extended Kalman filter

## Abstract

Reduced inertial sensor systems (RISS) have been introduced by many researchers as a low-cost, low-complexity sensor assembly that can be integrated with GPS to provide a robust integrated navigation system for land vehicles. In earlier works, the developed error models were simplified based on the assumption that the vehicle is mostly moving on a flat horizontal plane. Another limitation is the simplified estimation of the horizontal tilt angles, which is based on simple averaging of the accelerometers’ measurements without modelling their errors or tilt angle errors. In this paper, a new error model is developed for RISS that accounts for the effect of tilt angle errors and the accelerometer’s errors. Additionally, it also includes important terms in the system dynamic error model, which were ignored during the linearization process in earlier works. An augmented extended Kalman filter (EKF) is designed to incorporate tilt angle errors and transversal accelerometer errors. The new error model and the augmented EKF design are developed in a tightly-coupled RISS/GPS integrated navigation system. The proposed system was tested on real trajectories’ data under degraded GPS environments, and the results were compared to earlier works on RISS/GPS systems. The findings demonstrated that the proposed enhanced system introduced significant improvements in navigational performance.

## 1. Introduction

After its full operational capability was announced in 1995, the Global Positioning System (GPS) [1] became the dominant navigational system for land vehicles. Based on the principle of trilateration [2], a GPS receiver calculates its position by using range measurements from a minimum of four satellites [1,3]. Although GPS provides good long-term accuracy, there are a few drawbacks associated with it. Firstly, a GPS receiver needs radio signals from at least four satellites with a direct line-of-sight (LOS), which may not be possible in all scenarios. Secondly, a GPS signal might suffer from multipath or interference effects.

**Figure 1:**
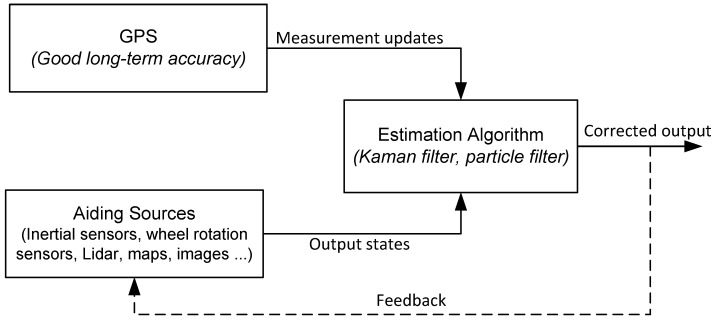
High level block diagram of system integration through estimation techniques.

To mitigate GPS errors, it can be integrated with other aiding sources with complementary characteristics using estimation algorithms, which are often based on a Kalman filter (KF) or a particle filter (PF). This integration results in superior accuracy compared to what any of the other systems can provide on their own [4,5,6,7,8,9]. A typical aiding source may consist of an inertial navigation system (INS) [10,11,12,13], speed sensors [14,15], light detection and ranging (LiDAR) [16,17,18], vision sensors [19,20,21], maps [22,23,24] *etc.* There are two major GPS/INS integration schemes, which are referred to as loosely-coupled (TC) or tightly-coupled (TC). The TC integration has the major advantage of providing an integrated solution even if satellite availability drops below four. There are two main variants of KF that are usually employed for GPS/INS integration, which are linearized KF (LKF) and extended KF (EKF). In LKF, the linearization is performed around a nominal trajectory (which is the output of a stand-alone INS), whereas in EKF, the linearization is always carried out around the corrected state, which keeps errors within the acceptable linearization range and performs better than LKF. A general block diagram of such an integrated system is shown in Figure 1.

With the advent of micro-electro-mechanical system (MEMS)-based technology, the cost of inertial sensors dropped significantly, which attracted many researchers to capitalize on this newer technology to develop accurate and economical navigation systems [13,25,26]. To reduce the effect of the complex error characteristics of MEMS-based inertial sensors and to avoid the higher cost of using all six inertial sensors of an inertial measurement unit (IMU), efforts were made to utilize fewer inertial sensors to compute the navigation solution [27]. This system is referred to as the reduced inertial sensor system (RISS), and its details can be found in [8,14,28,29].

Earlier works based on loosely-coupled LKF [28,30], tightly-coupled LKF [14] and PF [7,31] involving RISS/GPS integration suffer from limitations that adversely affect the accuracy of the system. For example, the system error models were simplified based on the assumption that the vehicle is mostly moving on a flat horizontal plane, and important terms in the system dynamic error model were ignored during the linearization process. Another limitation is the use of the simplified estimation of horizontal tilt angles. This was based on simple averaging of accelerometers without modelling accelerometer errors or tilt angles errors. The accelerometer biases were also not estimated in earlier approaches, which were based on LKF instead of EKF.

In this work, an improved EKF-based tightly-coupled RISS and GPS integration scheme is proposed, where an enhanced error model is developed, which considers additional important terms during the linearization of system model, leading to better positional accuracy. Furthermore, in the proposed work, the EKF is augmented to handle tilt (pitch and roll) angle errors and transversal accelerometer errors, taking the accelerometer readings as measurement updates. The proposed EKF-based RISS/GPS was tested on road test trajectories and compared to the two earlier systems that are based on a simplified RISS error model. The results show improved positional accuracy during long GPS outages, where the system maintained reliable accuracy by making use of the most recent sensor biases estimated by EKF.

## 2. The 3D Reduced Inertial Sensor System

**Figure 2:**
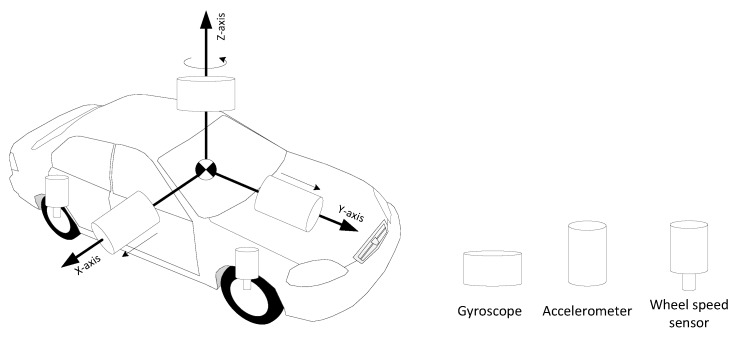
Arrangement of 3D reduced inertial sensor system (RISS) sensors with respect to the body frame.

The 3D RISS is a three-dimensional, low-cost reduced multi-sensor system consisting of two accelerometers mounted to the lateral and longitudinal directions of the vehicle frame and a vertically-aligned gyroscope in addition to the built-in vehicle speed sensor. The arrangement of these sensors with respect to the body frame is depicted in Figure 2.

The 3D RISS is very attractive in low-cost applications, because of its simplicity and elimination of two gyroscopes, which further reduces the overall cost and simplifies the modelling of complex error characteristics inherent in low-cost sensors. Furthermore, roll and pitch angles are obtained from accelerometers, which avoids mathematical integration, and position calculation involves only a single integration, which keeps the errors bounded.

**RISS Motion Equations** For the ensuing discussion and derivations, the local level reference frame is based on east, north and up (ENU) directions, and the x,y,z axes of the body are aligned with transversal, forward and vertically upward directions, respectively.

The rate of change of heading (azimuth) angle $\dot{A}$ is given as:

(1) $$\dot{A}=-\left[\left(\omega_{z}-b_{z}\right)-\omega^{e}\sin\text{φ}-\left(\frac{v_{e}\tan\text{φ}}{R_{N}+h}\right)\right]$$

where $\omega_{z}$ is the vertical gyroscope’s measurement, $b_{z}$ is the estimated gyroscope bias, $\omega^{e}$ is the Earth rotation rate, φ is the latitude, $v_{e}$ is the east velocity, *h* is the altitude of the vehicle and $R_{N}$ is the normal radius of the curvature of the Earth’s ellipsoid. It may be noted here that Equation (1) is different from the previous works on the subject [14,28,30,32], as the gyroscope’s measurement $\omega_{z}$ is being compensated for the bias $b_{z}$. This will also affect its linearization, as shown later in Section 3.1.4.

The computation of pitch and roll angles is based on the idea presented in [33,34]. Mathematically, the computation of the pitch angle is expressed as:

(2) $$p=\sin^{-1}\left(\frac{f_{y}-a_{o}}{g}\right)$$

where $f_{y}$ is the forward accelerometer measurement, $a_{o}$ is the vehicle acceleration ($a_{o}$) derived from the vehicle speed measurements and *g* is the Earth’s gravity.

The computation of the roll angle is given by:

(3) $$r=-\sin^{-1}\left[\frac{f_{x}+v_{o}\left(\omega_{z}-b_{z}\right)}{g\cos p}\right]$$

where $f_{x}$ is the transversal accelerometer measurement,$v_{o}$ is the vehicle speed obtained from wheel rotation sensor measurements and $\omega_{z}$ is the angular rate measured by the vertically-aligned gyroscope.

Once the azimuth and pitch angles are known, we can transform the vehicle’s speed along the forward direction $v_{o}$ to east, north and up velocities ($v_{e},v_{n}$ and $v_{u}$, respectively) according to the following expressions:

(4) $$v_{e}=v_{o}\sin A\cos p$$

(5) $$v_{n}=v_{o}\cos A\cos p$$

(6) $$v_{u}=v_{o}\sin p$$

The east and north velocities are transformed into geodetic coordinates and then integrated over the sample interval to obtain positions in latitude φ and longitude λ. The vertical component of velocity is integrated to obtain altitude *h*. These quantities are calculated using the following equations:

(7) $$\dot{\text{φ}}=\frac{v_{n}}{\left(R_{M}+h\right)}$$

(8) $$\dot{\text{λ}}=\frac{v_{e}}{(R_{N}+h)\cos\text{φ}}$$

(9) $$\dot{h}=v_{u}$$

where $R_{M}$ is the meridian radius of curvature and $R_{N}$ is the normal radius of the curvature of the Earth’s ellipsoid.

**Figure 3:**
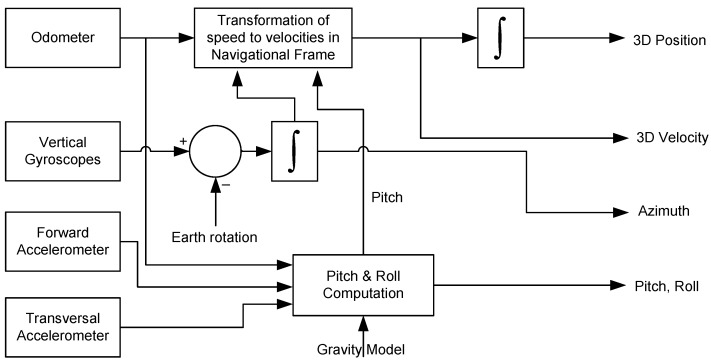
3D RISS mechanization block diagram.

A block diagram that illustrates the RISS mechanization system is shown in Figure 3.

A close look at Equations (1)–(6) reveals that the major source of errors in the RISS system is the error in the gyroscope measurements (gyroscope bias $b_{z}$), because it propagates to the azimuth, which propagates into an error in the horizontal channel velocity and position. Since no integration is involved in roll and pitch calculation in Equations (2) and (3), errors in the accelerometers’ measurements have a small effect.

## 3. Proposed EKF-Based 3D RISS/GPS Integration Algorithm

In the proposed algorithm, new system and measurement models were developed where the estimation of pitch and roll angles was included in EKF for better estimation of the attitude. Similarly, accelerometer bias estimation was performed through a filtering process. Furthermore, a new RISS error model was derived, which included additional important terms to improve the accuracy of the algorithm. The following sections will give the details of the system and measurement models for the proposed EKF-based integration of RISS and GPS.

### 3.1. System Model

The RISS error model is derived by linearization of the RISS mechanization Equations (1) and (4)–(9) use Taylor series expansion and retain only the first order terms. The error state vector of the 3D RISS is defined as follows:

(10) $$\begin{array}{cc} \delta x_{R}= & {\left[\delta \text{φ},\delta \text{λ},\delta h,\delta v_{e},\delta v_{n},\delta v_{u},\delta A,\delta a_{o},\delta b_{z},\delta r,\delta p,\delta b_{x},\delta b_{y},\delta v_{o}\right]}^{T} \end{array}$$

where δφ is latitude error, δλ is longitude error, δh is altitude error, $\delta v_{e}$ is east velocity error, $\delta v_{n}$ is north velocity error, $\delta v_{u}$ is upward velocity error, δA is azimuth error, $\delta a_{o}$ is error in acceleration derived from wheel rotation sensor measurements, $\delta b_{z}$ is gyroscope bias error, δr is error in roll, δp is error in pitch, $\delta b_{x}$ is bias error in lateral accelerometer, $\delta b_{y}$ is bias error in the forward accelerometer and $\delta v_{o}$ is error in forward velocity.

To get the rate of change of error states for the RISS system model, the motion equations of Section 2 have to be linearized through Taylor’s series expansion, and only the first order terms are retained. The error models for wheel rotation the sensor, gyroscope, accelerometers, pitch and roll angles are based on the first order Gauss–Markov processes. It is worth emphasizing here that the major contribution of this work lies in the treatment of velocities, azimuth and horizontal channel errors by augmenting EKF with additional error states. Therefore, their derivations will include some details where necessary. In order to facilitate the formulation of the final state transition matrix *F*, the position of the terms in the *F* matrix will be indicated by placing $F_{ab}$ under them. This indicates their position in the final *F* matrix, where subscript *a* denotes the row and *b* denotes the column.

#### 3.1.1. Latitude

The time rate of change of latitude φ is given in Equation (7), which is linearized using Taylor’s series as:

(11) $$\begin{array}{cc} \delta \dot{\text{φ}} & =\frac{\partial [.]}{\partial v_{n}}\delta v_{n}+\frac{\partial [.]}{\partial h}\delta h \end{array}$$

(12) $$\begin{array}{cc} \delta \dot{\text{φ}} & =\frac{\delta v_{n}}{\left(R_{M}+h\right)}-\frac{v_{n}\delta h}{{\left(R_{M}+h\right)}^{2}} \end{array}$$

since ${\left(R_{M}+h\right)}^{2}$ is a very large term, therefore:

(13) $$\begin{array}{cc} \delta \dot{\text{φ}} & \approx \underset{{\text{F}}_{15}}{\underset{︸}{\frac{1}{\left(R_{M}+h\right)}}}\delta v_{n} \end{array}$$

#### 3.1.2. Longitude

The time rate of change of longitude λ of Equation (8) is linearized through Taylor’s series as:

(14) $$\begin{array}{cc} \delta \dot{\text{λ}} & =\frac{\partial [.]}{\partial v_{e}}\delta v_{e}+\frac{\partial [.]}{\partial \text{φ}}\delta \text{φ}+\frac{\partial [.]}{\partial h}\delta h \end{array}$$

(15) $$\begin{array}{cc} \delta \dot{\text{λ}} & =\frac{\delta v_{e}}{(R_{N}+h)\cos\text{φ}}+\frac{v_{e}\sin\text{φ}\delta \text{φ}}{\left(R_{N}+h\right)\cos^{2}\text{φ}}-\frac{v_{e}\delta h}{{\left(R_{N}+h\right)}^{2}\cos\text{φ}} \end{array}$$

after ignoring the right most term due to ${\left(R_{M}+h\right)}^{2}$ being very large, we get:

(16) $$\begin{array}{cc} \delta \dot{\text{λ}} & \approx \underset{{\text{F}}_{24}}{\underset{︸}{\frac{1}{(R_{N}+h)\cos\text{φ}}}}\delta v_{e}+\underset{{\text{F}}_{21}}{\underset{︸}{\frac{v_{e}\tan\text{φ}}{(R_{N}+h)\cos\text{φ}}}}\delta \text{φ} \end{array}$$

#### 3.1.3. Altitude

The rate of change of altitude is directly related to the vertical velocity (Equation (9)), which can be linearized as:

(17) $$\begin{array}{cc} \delta \dot{h} & =\underset{{\text{F}}_{36}}{\underset{︸}{1}}\times \delta v_{u} \end{array}$$

#### 3.1.4. Attitude

The differential equation of azimuth angle (refer Equation (1)) can also be linearized using Taylor’s series as follows:

(18) $$\begin{array}{cc} \delta \dot{A} & =\frac{\partial [.]}{\partial b_{z}}\delta b_{z}+\frac{\partial [.]}{\partial \text{φ}}\delta \varphi +\frac{\partial [.]}{\partial v_{e}}\delta v_{e}+\frac{\partial [.]}{\partial h}\delta h \end{array}$$

we get:

(19) $$\begin{array}{cc} \delta \dot{A} & =-\left[-\delta b_{z}-\omega^{e}\cos\text{φ}\delta \text{φ}-\frac{v_{e}\sec^{2}\text{φ}\delta \text{φ}}{R_{N}+h}-\frac{\tan\text{φ}\delta v_{e}}{R_{N}+h}+\frac{v_{e}\tan\text{φ}\delta h}{{\left(R_{N}+h\right)}^{2}}\right] \end{array}$$

taking care of the minus sign and ignoring the right most term, which is negligible:

(20) $$\begin{array}{cc} \delta \dot{A} & \approx \underset{{\text{F}}_{79}}{\underset{︸}{1}}\times \delta b_{z}+\underset{{\text{F}}_{71}}{\underset{︸}{\left(\omega^{e}\cos\text{φ}+\frac{v_{e}\sec^{2}\text{φ}}{R_{N}+h}\right)}}\delta \text{φ}+\underset{{\text{F}}_{74}}{\underset{︸}{\frac{\tan\text{φ}}{R_{N}+h}}}\delta v_{e} \end{array}$$

#### 3.1.5. East Velocity

East velocity $v_{e}$ is computed from the vehicle’s forward velocity $v_{o}$ (obtained through the wheel rotation sensor) as given in Equation (4). We first take its derivative with respect to time as:

(21) $$\begin{array}{cc} {\dot{v}}_{e} & ={\dot{v}}_{o}\sin A\cos p+v_{o}\cos A\cos p\dot{A} \end{array}$$

substituting $a_{o}$ and $v_{n}$:

(22) $$\begin{array}{cc} {\dot{v}}_{e} & =a_{o}\sin A\cos p+v_{n}\dot{A} \end{array}$$

Now, linearizing the above equation using Taylor’s series:

(23) $$\begin{array}{cc} \delta {\dot{v}}_{e} & =\frac{\partial [.]}{\partial a_{o}}\delta a_{o}+\frac{\partial [.]}{\partial A}\delta A+\frac{\partial [.]}{\partial v_{n}}\delta v_{n}+\frac{\partial [.]}{\partial \dot{A}}\delta \dot{A} \end{array}$$

and after some mathematical manipulation, we finally obtain:

(24) $$\begin{array}{cc} \delta {\dot{v}}_{e} & =\underset{{\text{F}}_{48}}{\underset{︸}{\sin A\cos p}}\delta a_{o}+\underset{{\text{F}}_{47}}{\underset{︸}{a_{o}\cos A\cos p}}\delta A-\underset{{\text{F}}_{45}}{\underset{︸}{\left(\omega_{z}-b_{z}-\omega^{e}\sin\text{φ}-\frac{v_{e}\tan\text{φ}}{R_{N}+h}\right)}}\delta v_{n}+\underset{{\text{F}}_{49}}{\underset{︸}{v_{n}}}\delta b_{z}\\ & +\underset{{\text{F}}_{41}}{\underset{︸}{v_{n}\left(\omega^{e}\cos\text{φ}+\frac{v_{e}\sec^{2}\text{φ}}{R_{N}+h}\right)}}\delta \text{φ}+\underset{{\text{F}}_{44}}{\underset{︸}{\frac{v_{n}\tan\text{φ}}{R_{N}+h}}}\delta v_{e} \end{array}$$

#### 3.1.6. North Velocity

The vehicle’s north velocity $v_{n}$ is obtained from its forward velocity $v_{o}$, as depicted by Equation (5). Its time derivative is given as:

(25) $$\begin{array}{cc} {\dot{v}}_{n} & ={\dot{v}}_{o}\cos A\cos p-v_{o}\sin A\cos p\dot{A} \end{array}$$

Substituting $a_{o}$ and $v_{e}$:

(26) $$\begin{array}{cc} {\dot{v}}_{n} & =a_{o}\cos A\cos p-v_{e}\dot{A} \end{array}$$

Now, linearizing the above equation using Taylor’s series:

(27) $$\begin{array}{cc} \delta {\dot{v}}_{e} & =\frac{\partial [.]}{\partial a_{o}}\delta a_{o}+\frac{\partial [.]}{\partial A}\delta A+\frac{\partial [.]}{\partial v_{e}}\delta v_{e}+\frac{\partial [.]}{\partial \dot{A}}\delta \dot{A} \end{array}$$

and after some mathematically manipulation, we get the following form:

(28) $$\begin{array}{cc} \delta {\dot{v}}_{n} & =\underset{{\text{F}}_{58}}{\underset{︸}{\cos A\cos p}}\delta a_{o}-\underset{{\text{F}}_{57}}{\underset{︸}{a_{o}\sin A\cos p}}\delta A+\underset{{\text{F}}_{54}}{\underset{︸}{\left(\omega_{z}-b_{z}-\omega^{e}\sin\text{φ}-\frac{2v_{e}\tan\text{φ}}{R_{N}+h}\right)}}\delta v_{e}\underset{{\text{F}}_{59}}{\underset{︸}{-v_{e}}}\delta b_{z}\\ & -\underset{{\text{F}}_{51}}{\underset{︸}{v_{e}\left(\omega^{e}\cos\text{φ}+\frac{v_{e}\sec^{2}\text{φ}}{R_{N}+h}\right)}}\delta \text{φ} \end{array}$$

#### 3.1.7. Up Velocity

Vertical velocity is related to the forward velocity and pitch angle, as shown in Equation (6). First, we take the derivative with respect to time:

(29) $$\begin{array}{cc} {\dot{v}}_{u} & ={\dot{v}}_{o}\sin p \end{array}$$

(30) $$\begin{array}{cc} {\dot{v}}_{u} & =a_{o}\sin p \end{array}$$

Now, linearize the above equation using Taylor’s series:

(31) $$\begin{array}{cc} \delta {\dot{v}}_{u} & =\underset{{\text{F}}_{68}}{\underset{︸}{\sin p}}\delta a_{o} \end{array}$$

#### 3.1.8. Forward Velocity

The vehicle’s forward velocity is obtained from wheel rotation sensor measurements (odometer measurements), and the rate of change of its error is equal to the error in acceleration. This can be modelled as:

(32) $$\delta {\dot{v}}_{o}=\underset{F_{14,8}}{\underset{︸}{1}}\times \delta a_{o}$$

#### 3.1.9. Modelling of Horizontal Channel Errors

For land vehicles, roll and pitch angles have a limited range of values. Furthermore, in RISS, pitch and roll angles are computed using accelerometers instead of gyroscopes. Therefore, the errors in the horizontal channel are estimated through the use of first order Gauss–Markov processes as follows [35,36]:

(33) $$\begin{array}{cc} \delta \dot{r} & =-\beta_{r}\delta r+\sqrt{2\beta_{r}\sigma_{r}^{2}}w\left(t\right) \end{array}$$

(34) $$\begin{array}{cc} \delta \dot{p} & =-\beta_{p}\delta p+\sqrt{2\beta_{p}\sigma_{p}^{2}}w\left(t\right) \end{array}$$

(35) $$\begin{array}{cc} \delta {\dot{b}}_{x} & =-\beta_{x}\delta b_{x}+\sqrt{2\beta_{x}\sigma_{x}^{2}}w\left(t\right) \end{array}$$

(36) $$\begin{array}{cc} \delta {\dot{b}}_{y} & =-\beta_{z}\delta b_{y}+\sqrt{2\beta_{y}\sigma_{y}^{2}}w\left(t\right) \end{array}$$

where β and σ represent the reciprocal of the correlation times and standard deviations of the respective errors.

#### 3.1.10. Modelling of the Wheel Rotation Sensor and Gyroscope

The stochastic errors of the wheel rotation sensor and gyroscope are modelled by well-known first order Gauss–Markov processes.

(37) $$\begin{array}{cc} \delta {\dot{a}}_{o} & =-\beta_{o}\delta a_{o}+\sqrt{2\beta_{o}\sigma_{o}^{2}}w\left(t\right) \end{array}$$

(38) $$\begin{array}{cc} \delta {\dot{b}}_{z} & =-\beta_{z}\delta b_{z}+\sqrt{2\beta_{z}\sigma_{z}^{2}}w\left(t\right) \end{array}$$

where β and σ represent the reciprocal of the correlation times and standard deviations of the respective errors.

### 3.2. The GPS System Model for Tightly-Coupled RISS/GPS Integration

For tightly-coupled RISS/GPS integration, we have two additional states for the system model, which are GPS receiver clock bias $\delta b_{r}$ and its drift $\delta d_{r}$. The state vector for GPS error states is written as [14]:

(39) $$\delta x_{G}={\left[\delta b_{r},\delta d_{r}\right]}^{T}$$

The GPS receiver clock drift is modelled by a random walk process, and the receiver bias is the integral of the drift.

(40) $$\delta {\dot{b}}_{r}=\delta d_{r}$$

(41) $$\delta {\dot{d}}_{r}=\sigma_{d}w_{G}$$

where $w_{G}$ is white Gaussian noise and $\sigma_{d}$ is the standard deviation of white noise for clock drift.

Based on the equations of various errors states derived in Section 3.1, the complete dynamic matrix can be constructed as shown in Equation (42):

(42) $$F=\left[\begin{array}{cccccccccccccccc} 0 & 0 & 0 & 0 & F_{15} & 0 & 0 & 0 & 0 & 0 & 0 & 0 & 0 & 0 & 0 & 0\\ F_{21} & 0 & 0 & F_{24} & 0 & 0 & 0 & 0 & 0 & 0 & 0 & 0 & 0 & 0 & 0 & 0\\ 0 & 0 & 0 & 0 & 0 & 1 & 0 & 0 & 0 & 0 & 0 & 0 & 0 & 0 & 0 & 0\\ F_{41} & 0 & 0 & F_{44} & F_{45} & 0 & F_{47} & F_{48} & F_{49} & 0 & 0 & 0 & 0 & 0 & 0 & 0\\ F_{51} & 0 & 0 & F_{54} & 0 & 0 & F_{57} & F_{58} & F_{59} & 0 & 0 & 0 & 0 & 0 & 0 & 0\\ 0 & 0 & 0 & 0 & 0 & 0 & 0 & F_{68} & 0 & 0 & 0 & 0 & 0 & 0 & 0 & 0\\ F_{71} & 0 & 0 & F_{74} & 0 & 0 & 0 & 0 & 1 & 0 & 0 & 0 & 0 & 0 & 0 & 0\\ 0 & 0 & 0 & 0 & 0 & 0 & 0 & F_{88} & 0 & 0 & 0 & 0 & 0 & 0 & 0 & 0\\ 0 & 0 & 0 & 0 & 0 & 0 & 0 & 0 & F_{99} & 0 & 0 & 0 & 0 & 0 & 0 & 0\\ 0 & 0 & 0 & 0 & 0 & 0 & 0 & 0 & 0 & -\beta_{r} & 0 & 0 & 0 & 0 & 0 & 0\\ 0 & 0 & 0 & 0 & 0 & 0 & 0 & 0 & 0 & 0 & -\beta_{p} & 0 & 0 & 0 & 0 & 0\\ 0 & 0 & 0 & 0 & 0 & 0 & 0 & 0 & 0 & 0 & 0 & -\beta_{x} & 0 & 0 & 0 & 0\\ 0 & 0 & 0 & 0 & 0 & 0 & 0 & 0 & 0 & 0 & 0 & 0 & -\beta_{y} & 0 & 0 & 0\\ 0 & 0 & 0 & 0 & 0 & 0 & 0 & 1 & 0 & 0 & 0 & 0 & 0 & 0 & 0 & 0\\ 0 & 0 & 0 & 0 & 0 & 0 & 0 & 0 & 0 & 0 & 0 & 0 & 0 & 0 & 0 & 1\\ 0 & 0 & 0 & 0 & 0 & 0 & 0 & 0 & 0 & 0 & 0 & 0 & 0 & 0 & 0 & 0 \end{array}\right]$$

### 3.3. Measurements Model for Tightly-Coupled RISS/GPS Integration

The general nonlinear measurement model for KF is expressed as:

(43) z=h(x)+η

where z is the measurement vector and h(x) is the measurement design matrix, which relates the measurement vector with state vector x in a nonlinear fashion. The quantity **η** represents the measurements noise vector. Since KF works on linearized models, the linearized measurement model is given as:

(44) δz=Hδx+η

where δz is the error in the measurement vector, His the measurement design matrix and δx is the error state vector.

This work contains two sets of measurements that come from the GPS and two lateral accelerometers. Both of these are non-linearly related to the state vector; therefore, the next two sub-sections will address these models. The GPS measurement model has been detailed in earlier literature; therefore, only relevant details will be given, and the appropriate literature will be referenced. The accelerometer measurement model is a new contribution, which shall be derived in detail.

#### 3.3.1. GPS Measurement Updates

For the tightly-coupled integration, the measurement model is more involved, because the measurements are the difference in pseudoranges and pseudorange rates (instead of position and velocity), whereas the state vector consists of position, velocity, attitudes, *etc*. The measurement vector for *M* number of satellites is expressed as [14]:

(45) $$\delta z_{GPS}=\left[\begin{array}{c} {\text{ρ}}_{RSS}^{1}-{\text{ρ}}_{GPS}^{1}\\ {\text{ρ}}_{RSS}^{2}-{\text{ρ}}_{GPS}^{2}\\ \vdots \\ {\text{ρ}}_{RSS}^{M}-{\text{ρ}}_{GPS}^{M}\\ {\dot{\text{ρ}}}_{RSS}^{1}-{\dot{\text{ρ}}}_{GPS}^{1}\\ {\dot{\text{ρ}}}_{RSS}^{2}-{\dot{\text{ρ}}}_{GPS}^{2}\\ \vdots \\ {\dot{\text{ρ}}}_{RSS}^{M}-{\dot{\text{ρ}}}_{GPS}^{M} \end{array}\right]$$

where ${\text{ρ}}_{RSS}$, ${\dot{\text{ρ}}}_{RSS}$ are RISS estimated range and range rates and ${\text{ρ}}_{GPS}$, ${\dot{\text{ρ}}}_{GPS}$ are GPS measured range and range rates between the satellite and the receiver.

The *H* matrix of the overall GPS measurement model for the tightly-coupled integration with pseudorange and Doppler measurements from a total of *M* satellites will be of the following form [14]:

(46) $$\begin{array}{cc} & H_{GPS}=\left[\begin{array}{cccccc} H_{\text{ρ},M\times 3} & 0_{M\times 3} & 0_{M\times 3} & -1_{M\times 1} & 0_{M\times 1} & 0_{M\times 5}\\ 0_{M\times 3} & H_{\dot{\rho },M\times 3} & 0_{M\times 3} & 0_{M\times 1} & -1_{M\times 1} & 0_{M\times 5} \end{array}\right] \end{array}$$

where $H_{\text{ρ}}=G\times L$ and $H_{\dot{\rho }}=G\times R_{l}^{e}$. Here, *G* is the geometry matrix, *L* is a matrix, which represents the linearized form of the relationship that converts the geodetic position coordinates to ECEFCartesian coordinates, and $R_{l}^{e}$ is the transformation matrix from the local level frame to the ECEF frame. These matrices are defined as:

(47) $$G=\left[\begin{array}{ccc} 1_{x,RSS}^{1} & 1_{y,RSS}^{1} & 1_{z,RSS}^{1}\\ 1_{x,RSS}^{2} & 1_{y,RSS}^{2} & 1_{z,RSS}^{2}\\ \vdots \\ 1_{x,RSS}^{M} & 1_{y,RSS}^{M} & 1_{z,RSS}^{M} \end{array}\right]$$

where $1_{x,RSS}^{m}$ is the line of sight unit vector from the *m*-th satellite to the receiver position based on the output of RISS mechanization.

(48) $$L=\left[\begin{array}{ccc} -\left(R_{N}+h\right)\sin\text{φ}\cos\text{λ} & -\left(R_{N}+h\right)\cos\text{φ}\sin\text{λ} & \cos\text{φ}\cos\text{λ}\\ -\left(R_{N}+h\right)\sin\text{φ}\sin\text{λ} & +\left(R_{N}+h\right)\cos\text{φ}\cos\text{λ} & \cos\text{φ}\sin\text{λ}\\ \left[R_{N}\left(1-e^{2}\right)+h\right]\cos\text{φ} & 0 & \sin\text{φ} \end{array}\right]$$

(49) $$R_{l}^{e}=\left[\begin{array}{ccc} -\sin\text{λ} & -\sin\text{φ}\cos\text{λ} & \cos\text{φ}\cos\text{λ}\\ \cos\text{λ} & -\sin\text{φ}\sin\text{λ} & \cos\text{φ}\sin\text{λ}\\ 0 & \cos\text{φ} & \sin\text{φ} \end{array}\right]$$

#### 3.3.2. Horizontal Channel Measurement Updates

Apart from GPS, we also have measurements from forward accelerometer fy and lateral accelerometer fx, which constitute the horizontal channel. The measurement vector for the two accelerometers is expressed as:

(50) $$\delta z_{RSS}=\left[\begin{array}{c} f_{x,EST}-f_{x,OBS}\\ f_{y,EST}-f_{y,OBS} \end{array}\right]$$

where EST denotes the estimated quantity and OBS denotes the observed or measured value.

Since these measurements are not linearly related to the states, we need to linearize them, as well. This linearization is carried out next, where the terms below underbraces represent their place in design matrix $H_{RSS}$.

**Lateral Accelerometer** Assuming that the lateral accelerometer measurement has a bias $b_{x}$, it can be related to various states as follows:

(51) $$\begin{array}{cc} f_{x} & =-g\sin r\cos p-v_{o}\left(\omega_{z}-b_{z}\right)+b_{x} \end{array}$$

Linearizing the above equation using Taylor’s series:

(52) $$\begin{array}{cc} \delta f_{x} & =\frac{\partial [.]}{\partial r}\delta r+\frac{\partial [.]}{\partial p}\delta p+\frac{\partial [.]}{\partial v_{o}}\delta v_{o}+\frac{\partial [.]}{\partial b_{z}}\delta b_{z}+\frac{\partial [.]}{\partial b_{x}}\delta b_{x} \end{array}$$

we obtain the following:

(53) $$\begin{array}{cc} \delta f_{x} & =\underset{H_{1,12}}{\underset{︸}{-g\cos r\cos p}}\delta r+\underset{H_{1,13}}{\underset{︸}{g\sin r\sin p}}\delta p\underset{H_{1,16}}{\underset{︸}{-(\omega_{z}-b_{z})}}\delta v_{o}+\underset{H_{1,9}}{\underset{︸}{v_{o}}}\delta b_{z}+\underset{H_{1,14}}{\underset{︸}{\delta b_{x}}} \end{array}$$

**Forward Accelerometer** Similarly, assuming that the forward accelerometer measurement has a bias $b_{y}$, it can be related to various states as follows:

(54) $$\begin{array}{cc} f_{y} & =g\sin p+a_{o}+b_{y} \end{array}$$

Linearizing the above equation using Taylor’s series:

(55) $$\begin{array}{cc} \delta f_{y} & =\frac{\partial [.]}{\partial p}\delta p+\frac{\partial [.]}{\partial a_{o}}\delta a_{o}+\frac{\partial [.]}{\partial b_{y}}\delta b_{y} \end{array}$$

we obtain the following:

(56) $$\begin{array}{cc} \delta f_{y} & =\underset{H_{2,13}}{\underset{︸}{g\cos p}}\delta p+\underset{H_{1,8}}{\underset{︸}{\delta a_{o}}}+\underset{H_{2,15}}{\underset{︸}{\delta b_{y}}} \end{array}$$

In light of the above, the $H_{RSS}$ matrix of the measurement model for the two accelerometers can be written as:

(57) $$H_{RSS}=\left[\begin{array}{ccccccccc} 0_{1\times 7} & 0 & H_{1,9} & 0_{2\times 2} & H_{1,12} & H_{1,13} & H_{1,14} & 0 & H_{1,16}\\ 0_{1\times 7} & H_{2,8} & 0 & 0_{2\times 2} & 0 & H_{2,13} & 0 & H_{2,15} & 0 \end{array}\right]$$

The overall measurement model can be expressed as:

(58) $$\left[\begin{array}{c} \delta z_{GPS}\\ \delta z_{RSS} \end{array}\right]={\left[\begin{array}{c} H_{GPS}\\ H_{RSS} \end{array}\right]}_{(2M+2)\times 16}\delta x+\eta$$

**Figure 4:**
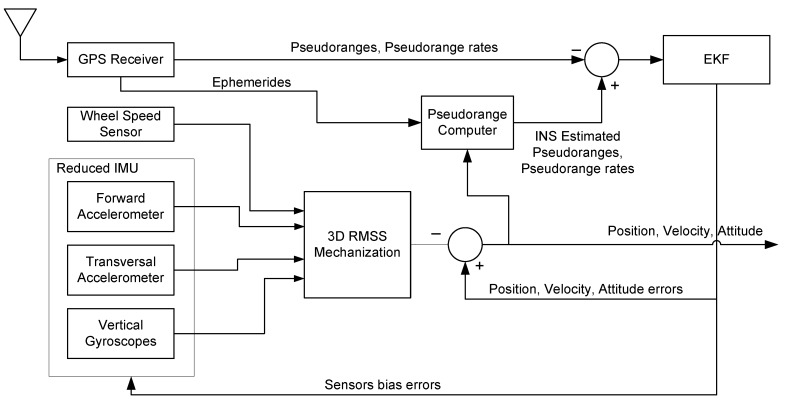
EKF-based RISS/GPS tightly-coupled integrated navigation system.

The Kalman filter and its variants are well known, and there is a great deal of literature that explains all of their details and nuances. Therefore, the details will not be repeated in this text. However, the reader is referred to some excellent material in [35,36,37,38]. The block diagrams of tightly-coupled integration with a closed-loop scheme are shown in Figure 4.

## 4. Experimental Work and Results

### 4.1. Equipment Setup

**Figure 5:**
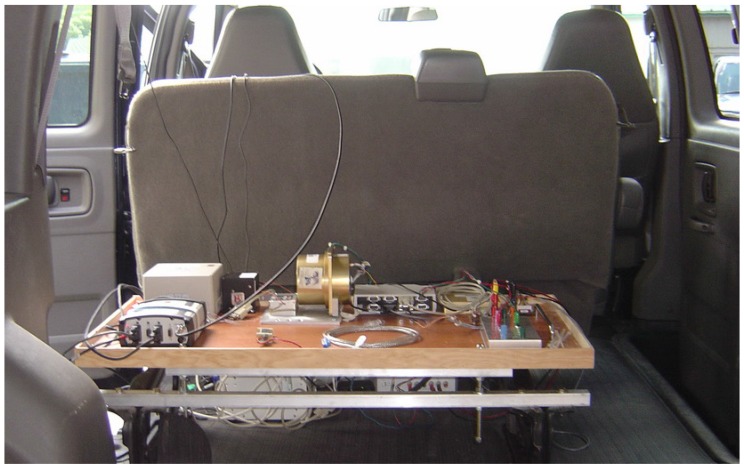
Navigation equipment and supporting hardware placed in a van for trajectory data collection.

The performance of the proposed improved tightly-coupled 3D RISS/GPS algorithm was examined on real road data using a low-cost MEMS-based Crossbow IMU300CC along with odometry obtained from the vehicle’s OBDIIinterface using the Carchip data logger. Figure 5 shows the equipment inside a van that was used to conduct the road experiments to collect the data.

The reference systems are based on the Honeywell HG1700 AG17 high-end tactical-grade IMU, which is tightly integrated with the NovAtel OEM4 GPS receiver using the OEM4-G2 ProPak-G2plus SPAN unit developed by NovAtel. Table 1 lists the major specification of the IMU300CC and HG1700 IMUs.

The results of two test trajectories are reported for this paper to show the performance of the proposed algorithm and for a comparison with earlier works. The selection of these trajectories was based on the criteria that they include driving conditions that a driver may encounter in a typical road trip through downtown, urban and suburban areas. The purpose of downtown driving, especially for the Toronto downtown trajectory, was to see the performance of the algorithm in severe multipath and poor GPS satellite visibility.

**Table 1:** Characteristics of the Crossbow and Honeywell IMU.

	Crossbow IMU300CC	HG1700
Size	7.62 × 9.53 × 8.13 cm	16 × 16 × 10 cm
Weight	0.59 kg	3.4 kg
Max data rate	200 Hz	100 Hz
Start-up time	<1 s	<0.8 s
**Accelerometer**		
Range	±2 g	±50 g
Bias	<± 30 mg	1 mg
Scale factor	<1 %	300 ppm
Random walk	<0.15 m/s/$\sqrt{\text{h}}$	<0.198 m/s/$\sqrt{\text{h}}$
**Gyroscope**		
Range	$\pm 100^{\circ }$/s	$\pm 1000^{\circ }$/s
Bias	$<\pm 2.0^{\circ }$/s	1${}^{\circ }$/h
Scale factor	<1%	150 ppm
Random walk	$<2.25^{\circ }/\sqrt{\text{h}}$	$<0.125^{\circ }/\sqrt{\text{h}}$

### 4.2. Evaluation and Comparison Criteria

The criteria for evaluating the algorithm were based on the maximum 2D position errors during the satellite blockages. The 2D positional accuracy of the proposed system (TC-EKF) is compared to earlier RISS/GPS integrated systems by quantifying the overall percentage improvement. These include loosely-coupled LKF (LC-LKF) [28] and loosely-coupled PF (LC-PF) [31], as well as tightly-coupled LKF (TC-LKF) [14]. When comparing the performance of the proposed system with other systems during GPS outages, we take the zero satellite case. In addition, comparative error plots shall be given when comparing the TC-LKF with the proposed TC-EKF for a more thorough examination of positional errors.

### 4.3. Kingston Downtown Trajectory

**Figure 6:**
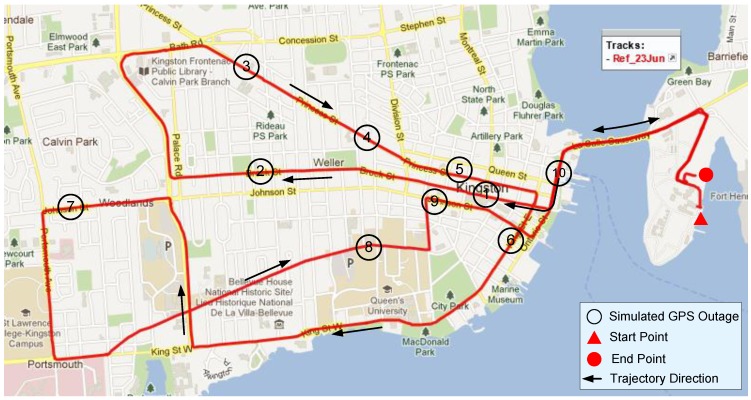
Test trajectory in Kingston downtown, ON, Canada.

This trajectory was conducted in the heart of Kingston city, and ten GPS signal outages were introduced at locations, such that they encompass all challenging driving scenarios, including low speeds, normal turns, sharp turns, high speeds and slopes, *etc*. The outage duration was fixed to 60 s, and every outage was repeated four times, where visible satellites were decreased from three to zero, one by one, while the response and accuracy of the algorithm was analysed. Figure 6 depicts this trajectory where outage locations are shown with black circles.

#### 4.3.1. Positional Errors

**Figure 7:**
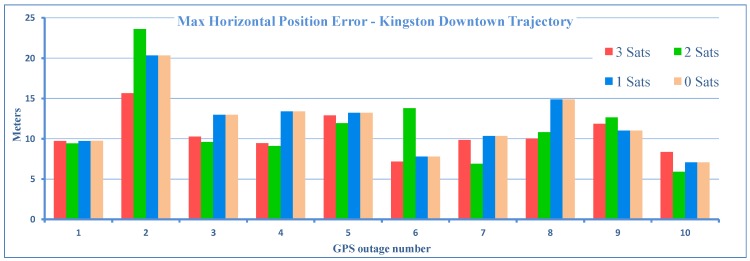
Maximum 2D positional errors for GPS outages of the Kingston downtown trajectory.

The resulting maximum horizontal position errors during the ten outages, each introduced by limiting the number of satellite to 3, 2, 1 and zero, are plotted as a bar graph in Figure 7.

**Figure 8:**
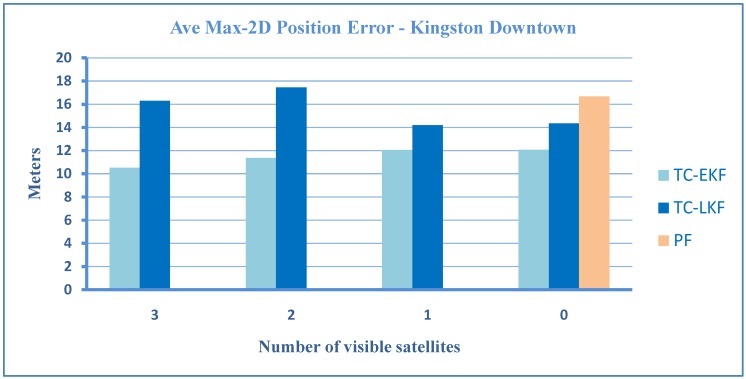
A comparison of tightly-coupled (TC-EKF), TC-linearized KF (LKF) and particle filter (PF) for the average maximum 2D position error for the Kingston downtown trajectory.

**Figure 9:**
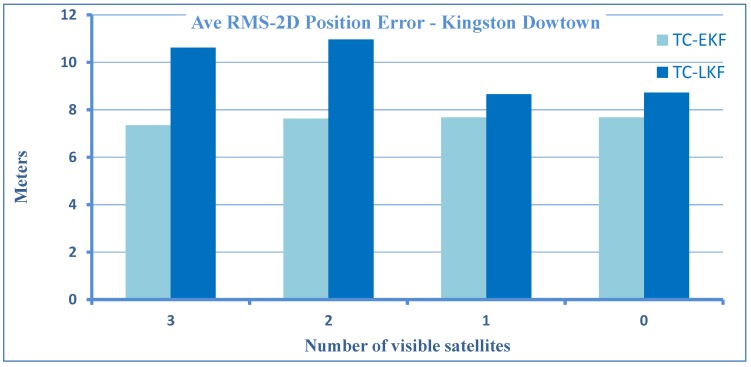
A comparison of TC-EKF with TC-LKF for the average RMS 2D position error for the Kingston downtown trajectory.

Here, it is important to note that, although, in general, the errors increase when the number of satellites decreases, however, in some outages, the addition of satellites actually increases the error. This happens when a satellite is at a low elevation; its signal is severely affected by atmospheric errors, and it is more prone to produce multipath effects [39]. Therefore, when this satellite is added to available satellites, the accuracy will decrease instead of improving. We also notice that in some cases, zero and one available satellite show better performance than two or three. This can happen if the external sensor aiding (inertial and wheel rotation sensors) is better due to reduced sensors and a superior algorithm, which accurately estimates sensor errors. This shows that that the algorithm can handle the partial, as well as total GPS signal blockage very well. Taking the zero satellite case as a reference, there is an improvement of 57% over LC-LKF [28], 16% over TC-LKF [14] and 28% over PF [31] of earlier works. A comparison of proposed TC-EKF, TC-LKF of [14] and PF of [31] is given in the bar plots of Figure 8 for the overall maximum horizontal positional errors. It may be noted that for the case of PF, only the zero satellite case can be compared, as it is based on a loosely-coupled architecture. It is evident that TC-EKF performs better then TC-LKF for all cases (3, 2, 1 and 0 satellites), and on average, the maximum position error is always less then 12 m, irrespective of the number of visible satellites. Figure 9 shows a similar comparison for the overall RMS horizontal positional errors. PF could not be included in this comparison, as it was not reported in the PF paper [31].

Table 2 summarizes the RMS errors for 3D position, attitude, as well as velocities during all of the full outages (zero available satellite) for the Kingston downtown trajectory.

**Table 2:** RMS errors during full 60-second outages for the Kingston downtown trajectory.

Outage No.	Position (m)				Attitude (${}^{\circ }$)				Velocity (m/s)		
	Lat	Long	Alt		Pitch	Roll	Azi		Ve	Vn	Vu
1	4.46	2.42	3.71		1.73	0.70	1.17		0.55	0.48	1.12
2	12.54	3.99	4.76		1.73	0.55	1.48		0.59	0.75	1.20
3	2.86	8.12	4.50		1.74	0.48	0.37		0.74	0.33	0.62
4	2.35	7.26	5.12		1.88	0.31	0.33		0.67	0.23	0.92
5	7.55	6.67	1.78		2.84	0.20	1.15		0.77	0.20	0.44
6	4.70	1.60	1.36		2.51	0.31	0.28		0.36	0.57	0.87
7	2.27	4.17	4.65		1.31	0.58	1.80		0.67	0.56	1.00
8	7.70	8.25	4.83		1.65	0.35	0.90		0.49	0.26	0.57
9	6.14	1.32	1.70		1.37	0.53	1.41		0.73	0.57	0.70
10	4.24	2.70	0.76		1.68	0.59	0.47		0.19	0.41	0.39
Average	5.48	4.65	3.32		1.84	0.46	0.94		0.58	0.44	0.78

**Figure 10:**
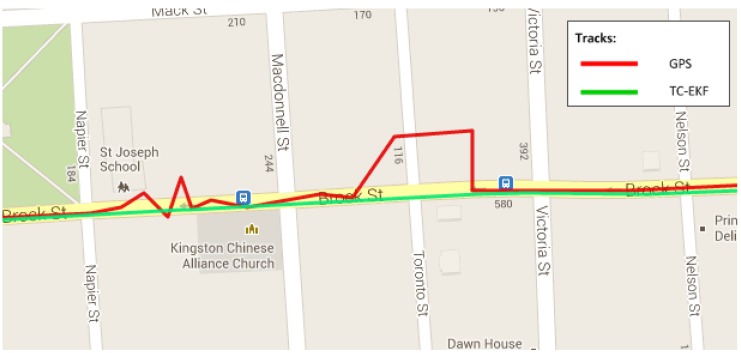
Noisy GPS during the Kingston downtown trajectory.

To show the robustness of the RISS system and the ability to reject or mitigate positional errors due to multipath or degraded GPS signals, a portion of the trajectory is shown in Figure 10 where GPS updates (in red) are totally unreliable (most likely due to multipath effects). The figure clearly shows the efficacy of the proposed TC-EKF system (in green) in mitigating the positional inaccuracy during the wrong GPS updates by keeping the trajectory on the road instead of following the erratic GPS measurements.

#### 4.3.2. Tilt Angle Errors

**Figure 11:**
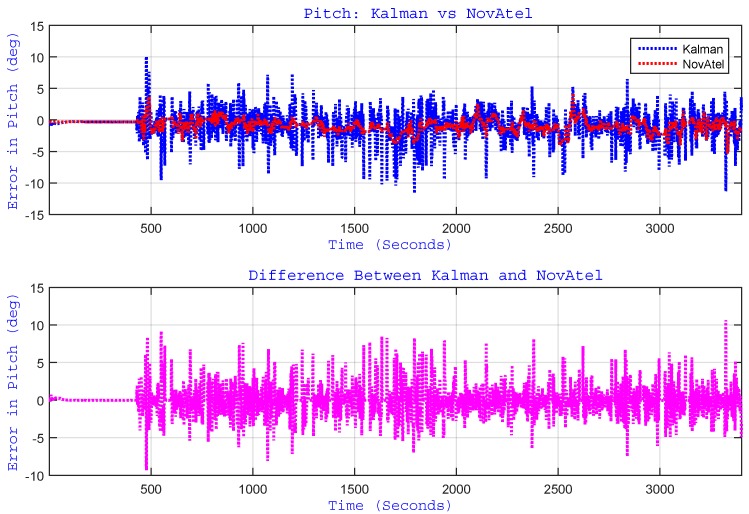
(Top) Performance of estimated pitch angle *versus* the reference (NovAtel) for the Kingston trajectory. The bottom plot shows the difference between the two systems.

**Figure 12:**
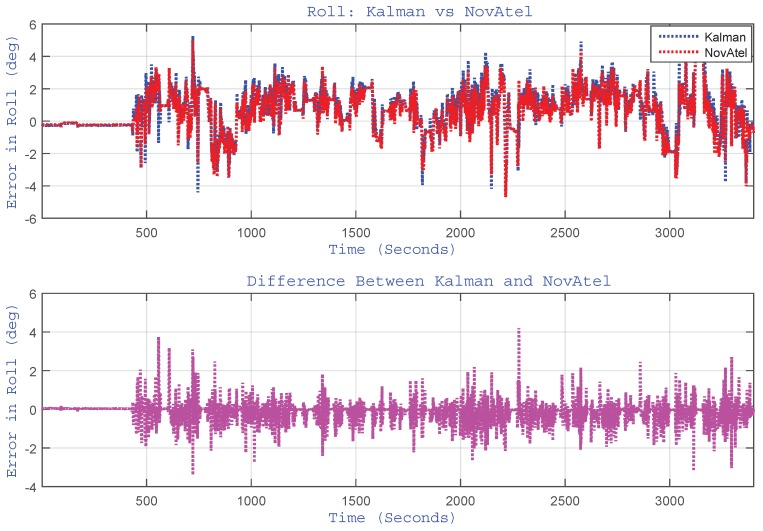
(Top) Performance of estimated roll angle *versus* the reference (NovAtel) for the Kingston trajectory. The bottom plot shows the difference between the two systems.

As indicated earlier, the proposed algorithm updates the pitch and roll angles by estimating accelerometer biases through EKF. Although the pitch and roll angles are very small for a land vehicle application, they are reported here to have a feel of their trend and to show the performance of the algorithm and its limitations. The plots of pitch and roll angles for the whole trajectory are shown in Figure 11 and Figure 12, respectively. These figures show the performance of the proposed algorithm (labelled as “Kalman”) with respect to the reference (labelled as “NovAtel”). They also show the plot of the difference (error) between the two systems. For this trajectory, the RMS for the pitch angle was 1.82${}^{\circ }$, and for roll angle, it was 0.52${}^{\circ }$.

It can be observed from Figure 11 that the pitch angle estimation is relatively noisy. This is due to the fact that its computation involves the differentiation of wheel rotation sensor measurements, which resulted in an increased noise level. This is one of the limitations of our method, which calls for future research.

#### 4.3.3. Gyroscope Bias

**Figure 13:**
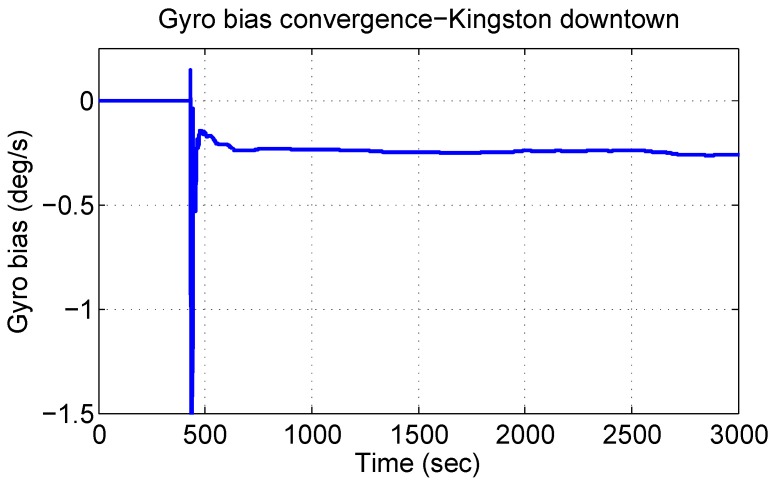
Gyroscope bias estimation convergence in the Kingston downtown trajectory.

As described earlier, the proposed system developed a new approach to estimate the gyroscope bias, which helps to improve the navigational accuracy during GPS outages. The convergence of this bias to its actual value is an important test for the algorithm and crucial to positional accuracy. Figure 13 depicts the the behaviour of the gyroscope bias convergence where its estimate converged to its true bias value of ≈−0.24${}^{\circ }$/s. The convergence starts with motion and takes almost 300 s to converge. A similar behaviour and the convergence value is shown later for the other three trajectories, as well, proving the repeatability of the algorithm.

### 4.4. Toronto Downtown Trajectory

**Figure 14:**
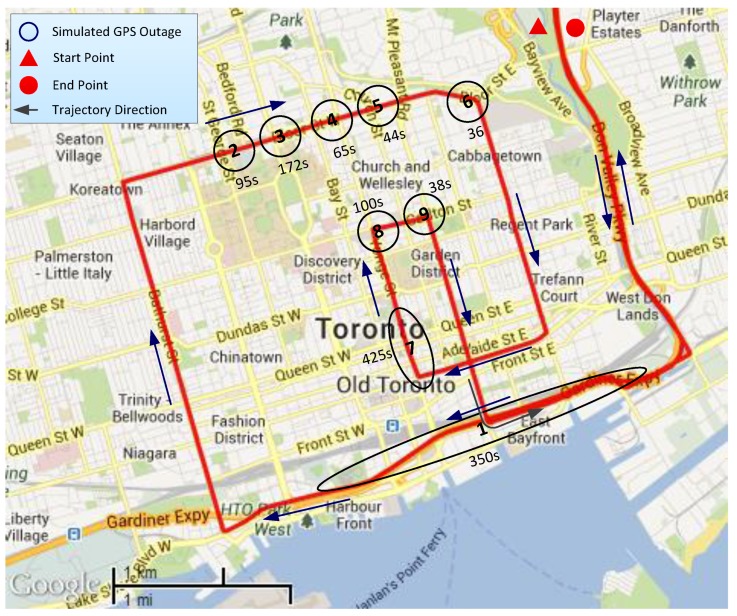
Main section of the test trajectory in downtown Toronto, ON, Canada.

To show the performance of the proposed algorithm in severe natural GPS outages, the Toronto downtown trajectory was selected. The trajectory lasted for about 90 min, and the main portion of the trajectory is depicted in Figure 14, where natural outages are marked with black circles/ellipses and their duration is written beside each outage. This trajectory is particularly challenging, as the test vehicle was driven through the heart of this metropolitan city, which has very tall buildings, tight turns, bridges and long overpasses, which severely restrict the availability of the visible satellite and also generate strong multipath signals.

**Figure 15:**
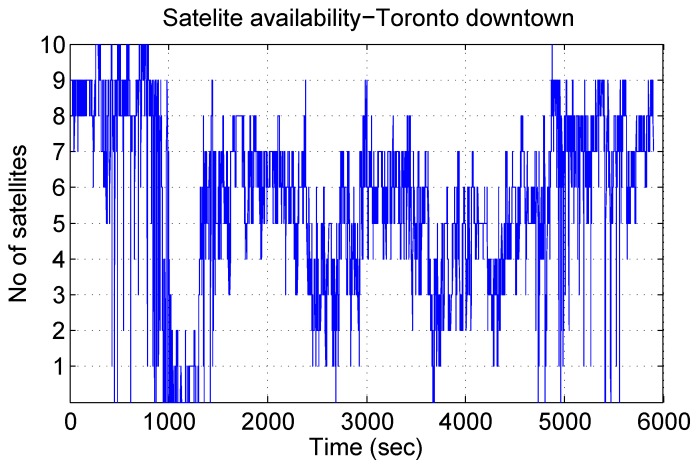
Availability of the satellites during the Toronto downtown trajectory.

Figure 15 gives an idea about the limited number of GPS satellites that were visible during the trajectory. Except for the initial portion and a couple of other places, most of the trajectory sees less than four satellites, and in many cases, the visible satellites even drop to zero. In this condition, we cannot take the earlier approach, where we reduced the number of available satellites from four gradually to zero in post-processing; rather, we pick the places of the natural outages and compare these to the results of earlier works.

#### 4.4.1. Positional Errors

**Figure 16:**
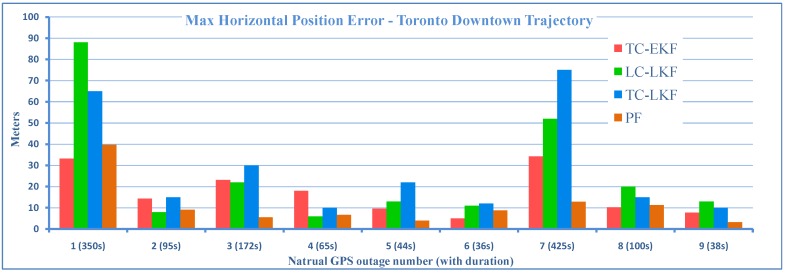
Maximum 2D positional errors of TC-EKF, the LC-LKF of [28], the TC-LKF of [14] and the PF of [31].

The 2D maximum positional errors of the proposed work during nine natural GPS outages of varying duration are compared to three of the earlier works. Figure 16 shows the comparison of the results with the LC-LKF of [28], the TC-LKF of [14] and the PF of [31] for all nine natural GPS outages of different durations. It can be seen that except for a couple of outages, the proposed TC-EKF performed better then both of the competitors based on KF. On average, TC-EKF outperformed LC-LKF by 50% and TC-LKF by 63%. As compared to PF [31], TC-EKF performed 35% poorer; however, it may be noted that KF still has the advantage of being computationally less expensive.

Table 3 lists the RMS errors for 3D position, attitude, as well as velocities during all of the natural outages for the Toronto downtown trajectory.

**Table 3:** RMS errors during full outages for the Toronto downtown trajectory.

Outage No.	Outage Duration	Position (m)				Attitude (deg)				Velocity (m/s)		
		Lat	Long	Alt		Pitch	Roll	Azi		Ve	Vn	Vu
1	350	9.05	14.24	12.18		3.17	0.61	2.31		1.28	1.92	1.94
2	95	8.34	5.18	3.16		3.05	0.35	1.99		0.62	0.58	0.98
3	172	15.77	6.10	10.60		2.94	0.32	3.80		0.64	0.41	0.55
4	65	7.77	11.78	42.20		1.63	0.14	2.08		0.34	0.10	0.35
5	44	7.82	2.53	5.35		4.34	0.23	1.28		0.64	0.24	1.06
6	36	2.44	1.64	4.73		3.59	0.86	4.18		1.97	1.76	2.04
7	425	9.42	14.37	9.70		3.43	0.41	3.56		0.76	0.75	0.94
8	100	5.36	2.04	3.54		4.28	0.96	48.79		1.24	1.40	1.58
9	38	1.83	6.36	4.25		3.26	1.41	7.59		1.49	2.13	0.32
Average	147	7.53	7.14	10.63		3.30	0.59	8.40		1.00	1.03	1.08

**Figure 17:**
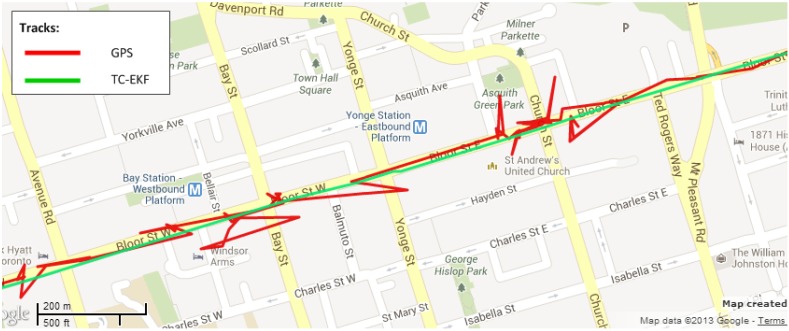
Performance in noisy GPS signal conditions during outage # 2, 3 and 4.

**Figure 18:**
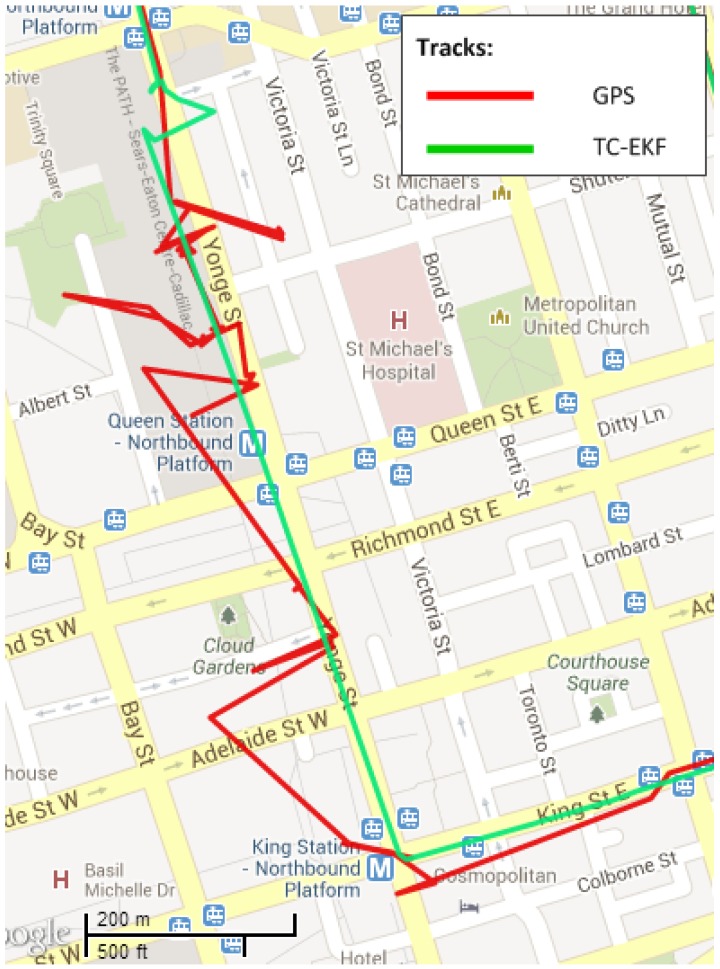
Performance in noisy and jeopardized GPS signal conditions during outage #7.

The Toronto downtown trajectory contains many signal multipath phenomena, which typically result in huge positional error. To show the performance of the proposed scheme in these environments, two sections of the trajectory are chosen; the first one encompasses outage Numbers 2, 3 and 4 and is shown in Figure 17; the second section contains outage Number 7, which is shown in Figure 18. In these examples, the GPS signal (in green) is going haywire, which is totally untrustworthy. However, the proposed algorithm (in red) is not affected by these jumps in GPS signal and stays very close to the road even in these severe environments.

#### 4.4.2. Tilt Angle Errors

**Figure 19:**
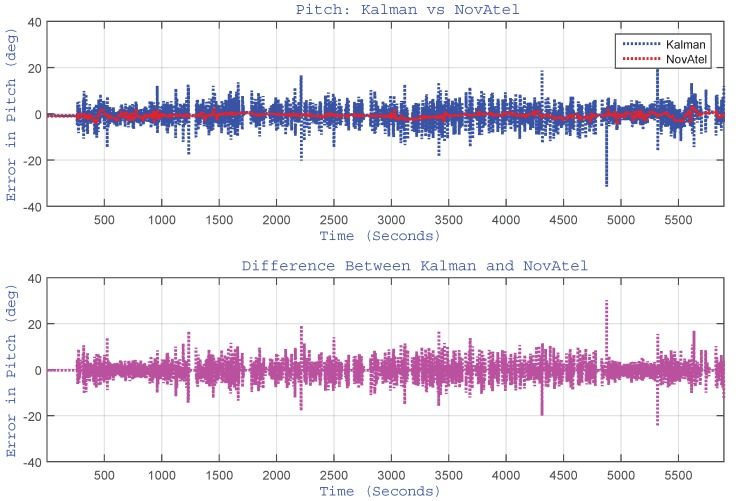
(Top) Performance of estimated pitch angle *versus* the reference for the Toronto trajectory. The bottom plot shows the difference between the two systems.

**Figure 20:**
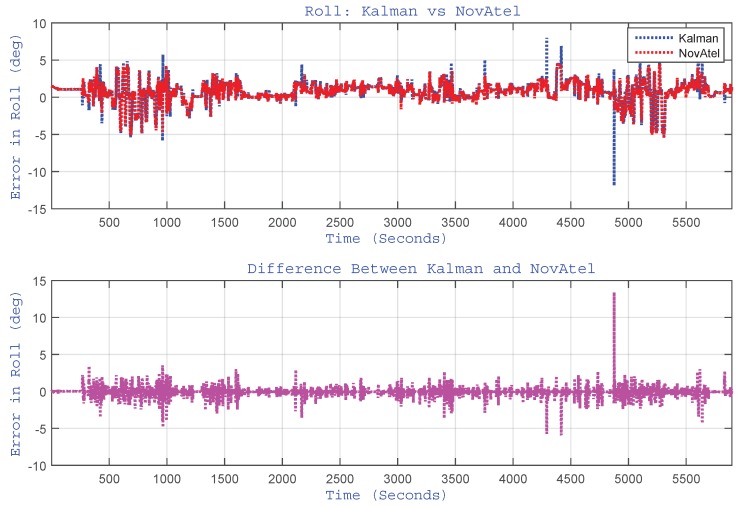
(Top) Performance of estimated roll angle *versus* the reference for the Toronto trajectory. The bottom plot shows the difference between the two systems.

The plots of pitch and roll angles for the Toronto trajectory are shown in Figure 19 and Figure 20, respectively. These figures show the performance of the proposed algorithm (labelled as “Kalman”) with respect to the reference (labelled as “NovAtel”) and the difference between them. For this trajectory, the RMS for the pitch angles was 3.11${}^{\circ }$, and for roll angle, it was 0.64${}^{\circ }$.

Here, also, it can be noticed from Figure 19 that the pitch angle estimation is relatively noisy for the same reason as highlighted earlier for the Kingston trajectory.

After incorporating the correction of pitch and roll angles (through the estimation of corresponding accelerometers biases), we noticed no appreciable improvement in pitch and roll angles. Although there is no tangible difference in pitch/roll estimation accuracy using the KF-based approach, the KF-based design enables the system to accept external roll/pitch updates (if/when available). This has the advantage of having the roll/pitch angle estimation within the filter design and processed in the main-stream of the KF algorithm, which makes the filter expandable for future design.

#### 4.4.3. Gyroscope Bias

**Figure 21:**
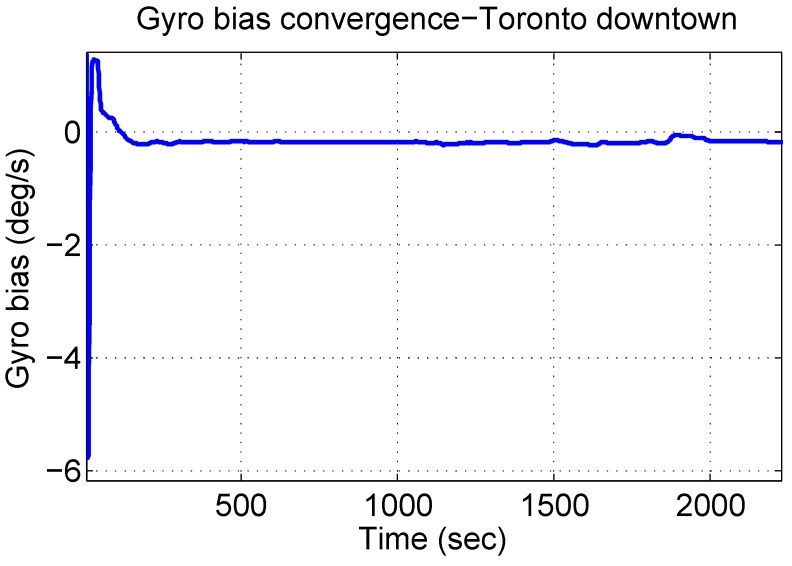
Convergence of gyroscope bias during the Toronto downtown trajectory.

It is interesting to see the convergence of the gyroscope bias value during this challenging trajectory, because the GPS updates are really noisy, and at times, there is no GPS signal at all, as already seen in Figure 15. Figure 21 shows the convergence of gyroscope bias during this trajectory, which successfully converges close to its actual values and stays smooth and steady. This trend helps the algorithm to stay close to the reference during GPS outages and noisy GPS updates.

## 5. Conclusions

This paper introduced an EKF-based 3D RISS/GPS integrated system where a new RISS dynamic error model was introduced, which models the effect of tilt angel errors and accelerometer errors. Furthermore, it included important terms in the system dynamic error model previously ignored during the linearization process in earlier works on RISS. As opposed to the earlier LKF-based algorithms, this proposed scheme uses EKF for RISS/GPS integration, where the RISS navigation output is corrected by EKF-estimated errors, which continuously keeps the linearization within an acceptable, accurate range, keeping the errors bounded. Road test results showed that the proposed EKF-based RISS/GPS system with the new error model outperforms the loosely-coupled LKF-based and tightly-coupled LKF-based RISS/GPS systems (with the over-approximated error model), providing more sustainable performance during long GPS outages. The proposed system demonstrated more accurate estimation of navigation state errors and sensor biases. In general, the proposed integration scheme showed higher accuracy, robustness and repeatability, outperforming the loosely-coupled LKF-based RISS/GPS integration by 53% and the tightly-coupled LKF-based RISS/GPS integration by 40% in overall maximum 2D positional accuracy. It showed slightly (4%) poorer performance than the loosely-coupled PF-based RISS/GPS. However, it may be noticed that KF has the advantage of lower computational burden over PF, which is crucial for real-time applications. Based on the superior results and use of low-cost sensors, the proposed navigation system is very promising for land vehicle navigation, as well as robotic applications.
